# Supplementary material for: A Type of Ferrocene-Based Derivative FE-1 COF Material for Glycopeptide and Phosphopeptide Selective Enrichment
Source: J Funct Biomater. 2024 Jul 4;15(7):185. doi: 10.3390/jfb15070185 (PMC11277842; doi:10.3390/jfb15070185)
Supplement: Supplementary file 1 [file jfb-15-00185-s001.zip › jfb-2967872-supplementary.pdf]

Table S1 Detailed information of phosphopeptides identified by MALDI-TOF MS in α-casein enzymatic solution after material enrichment

No.	m/z	Peptide Sequence	Number of phosphoryl groups
α1	1195.63	KNMAINP[pS]KENL	1
α2	1237.74	TVDME[pS]TEVF	1
α3	1253.55	TVD[Mo]E[pS]TEVF	2
α4	1337.39	HIQKEDV[pS]ER	1
α5	1466.57	TVDME[pS]TEVFIK	1
α6	1482.98	TVD[Mo]E[pS]TEVFTK	1
α7	1539.23	EQL[pS]T[pS]EENSKK	2
α8	1561.44	RELEELNVPGEIVE[pS]L[pS][pS][pS]EESITR	4
α9	1660.76	VPQLEIVPN[pS]AEER	1
α10	1832.58	YLGEYLIVPN[pS]AEER	1
α11	1927.37	DIG[pS]E[pS]TEDQAMEDIK	2
α12	1943.21	DIG[pS]E[pS]TEDQA[Mo]EDIK	2
α13	1951.46	YKVPQLEIVPN[pS]AEER	1
α14	2634.89	NT[Mo]EHV[pS][pS][pS]EE[pS]IISQETYK	4
α15	2703.53	Q*MEAE[pS][pS][pS][pS]EEIVPN[pS]VEAQK	5
α16	2720.64	QMEAE[pS][pS][pS][pS]EEIVPN[pS]VEAQK	5
α17	2736.73	Q[Mo]EAE[pS][pS][pS][pS]EEIVPN[pS]VEAQK	5
α18	3007.68	NANEEEYSIG[pS][pS][pS]EE[pS]AEVATEEVK	4

p: phosphorylation site. [Mo]: Oxidation of methionine residues

Table S2 Detailed information of phosphopeptides identified by MALDI-TOF MS in β-casein digest.

No.	m/z	Peptide Sequence	Number of phosphoryl groups
β1s	1031.40	FQ[pS]EEQQQTEDELQDK	1
β2s	1279.00	FQ[pS]EEQQQTEDELQDKIHPF	1
β3m	1561.55	RELEELNVPGEIVE[pS]L[pS][pS][pS]EESITRI	4
β4s	2061.76	FQ[pS]EEQQQTEDELQDK	1
β5m	2352.75	NVPGEIVESL[pS][pS][pS]EE[pS]ITR	4
β6s	2555.99	FQ[pS]EEQQQTEDELQDKIHPF	1
β7m	2886.60	ELEELNVPGEIVESLS[pS][pS]EESITR	2
β8m	3122.16	RELEELNVPGEIVE[pS]L[pS][pS][pS]EESITR	4

p: phosphorylation site. [Mo]: Oxidation of methionine residues

Table S3 Detailed information of glycopeptides identified by MALDI-TOF MS in IgG enzymatic solution

No.	m/z	Glycan composition	Glycopeptide sequence
I1	2269.67	[Hex]3[HexNAc]2[Fuc]1	EEQYN#STYR
I2	2399.78	[Hex]3[HexNAc]3[Fuc]1	EEQFN#STFR
I3	2430.89	[Hex]3[HexNAc]3[Fuc]1	EEQYN#STYR
I4	2560.49	[Hex]4[HexNAc]3[Fuc]1	EEQFN#STFR
I5	2601.37	[Hex]3[HexNAc]4[Fuc]1	EEQFN#STFR
I6	2617.81	[Hex]4[HexNAc]4	EEQFN#STFR
I7	2633.73	[Hex]3[HexNAc]4[Fuc]1 or [Hex]4[HexNAc]4	EEQYN#STYR or EEQFN#STYR
I8	2649.42	[Hex]3[HexNAc]4[Fuc]1	EEQYN#STYR
I9	2763.84	[Hex]4[HexNAc]4[Fuc]1	EEQFN#STFR
I10	2780.95	[Hex]5[HexNAc]4 or [Hex]4[HexNAc]4[Fuc]1	EEQFN#STFR or EEQFN#STYR
I11	2796.47	[Hex]4[HexNAc]4[Fuc]1 or [Hex]5[HexNAc]4	EEQYN#STYR or EEQFN#STYR
I12	2805.19	[Hex]3[HexNAc]5[Fuc]1	EEQFN#STFR
I13	2811.38	[Hex]5[HexNAc]4	EEQYN#STYR
I14	2837.54	[Hex]3[HexNAc]5[Fuc]1 or [Hex]4[HexNAc]5	EEQYN#STYR or EEQFN#STYR
I15	2925.63	[Hex]5[HexNAc]4[Fuc]1	EEQFN#STFR
I16	2943.59	[Hex]5[HexNAc]4[Fuc]1	EEQFN#STYR
I17	2958.71	[Hex]5[HexNAc]4[Fuc]1	EEQYN#STYR
I18	2966.97	[Hex]4[HexNAc]5[Fuc]1	EEQFN#STFR
I19	2999.45	[Hex]4[HexNAc]5[Fuc]1 or [Hex]5[HexNAc]5	EEQYN#STYR or EEQFN#STYR
I20	3129.36	[Hex]5[HexNAc]5[Fuc]1	EEQFN#STFR
I21	3161.58	[Hex]5[HexNAc]5[Fuc]1	EEQYN#STYR

N# Indicates the detected N-glycosylated peptide sites.

Table S4 Detailed information of glycopeptides and phosphopeptides detected after enrichment of IgG digest and α-casein enzymatic solution

No.	m/z	Glycan composition	Glycopeptide sequence
I1	2236.6	[Hex]3[HexNAc]2[Fuc]1	EEQFN#STFR
I2	2268.6	[Hex]3[HexNAc]2[Fuc]1	EEQYN#STYR
I3	2399.67	[Hex]3[HexNAc]3[Fuc]1	EEQFN#STFR
I4	2487.54	[Hex]3[HexNAc]3[Fuc]1	EEQYN#STYR
I5	2560.97	[Hex]4[HexNAc]3[Fuc]1	EEQFN#STFR
I6	2601.64	[Hex]3[HexNAc]4[Fuc]1	EEQFN#STFR
I7	2633.60	[Hex]3[HexNAc]4[Fuc]1 or [Hex]4[HexNAc]4	EEQYN#STYR or EEQFN#STYR
I8	2642.63	[Hex]3[HexNAc]5	EEQFN#STFR
I9	2763.65	[Hex]4[HexNAc]4[Fuc]1	EEQFN#STFR
I10	2795.64	[Hex]4[HexNAc]4[Fuc]1 or [Hex]5[HexNAc]4	EEQYN#STYR or EEQFN#STYR
I11	2852.64	[Hex]4[HexNAc]5	EEQYN#STYR
I12	2925.66	[Hex]5[HexNAc]4[Fuc]1	EEQFN#STFR
I13	2958.60	[Hex]5[HexNAc]4[Fuc]1	EEQYN#STYR
I14	2998.66	[Hex]4[HexNAc]5[Fuc]1	EEQYN#STYR
I15	3054.88	[Hex]4[HexNAc]4[Fuc]1[NeuAc]1	EEQFN#STYR
I16	3088.23	[Hex]4[HexNAc]4[Fuc]1[NeuAc]1	EEQYN#STFR
I17	3129.04	[Hex]5[HexNAc]5[Fuc]1	EEQFN#STFR
I18	3245.60	[Hex]4[HexNAc]4[Fuc]1	TKPREEQFN#STFR
I19	3279.72	[Hex]4[HexNAc]4[Fuc]1	TKPREEQYN#STYR

N# Indicates the detected N-glycosylated peptide sites.

No.	m/z	Peptide Sequence	Number of phosphoryl groups
α1	1124.1	KEKVNEL[pS]KDIG[pS]E[pS]TEDQA	3
α2	1253.30	TVD[Mo]E[pS]TEVF	2
α3	1337.56	HIQKEDV[pS]ER	1
α4	1466.44	TVDME[pS]TEVFIK	1
α5	1482.53	TVD[Mo]E[pS]TEVFTK	1
α7	1539.43	EQL[pS]T[pS]EENSKK	2
α8	1634	ED[pS]PEVIESPPEIN	1
α9	1660.68	VPQLEIVPN[pS]AEER	1
α10	1832.75	YLGEYLIVPN[pS]AEER	1
α11	1927.54	DIG[pS]E[pS]TEDQAMEDIK	2
α12	1943.53	DIG[pS]E[pS]TEDQA[Mo]EDIK	2
α13	1951.84	YKVPQLEIVPN[pS]AEER	1
α14	2079.88	KKYKVPQLEIVPN[pS]AEER	4
α15	2337.07	VPQLEIVPN[pS]AEERLH[pS]MK	2
α16	3007.82	NANEEEYSIG[pS][pS][pS]EE[pS]AEVATEEVK	4

p: phosphorylation site. [Mo]: Oxidation of methionine residues

Table S5 Detailed information of phosphopeptides detected after enrichment of human serum

No.	m/z	Peptide sequence	Number of phosphoryl groups
HS1	1389.32	D[pS]GEGDFLAEGGGV	1
HS2	1460.36	AD[pS]GEGDFLAEGGGV	1
HS3	1545.43	D[pS]GEGDFLAEGGGVR	1
HS4	1616.46	AD[pS]GEGDFLAEGGGVR	1

Table S6 Detailed information of glycopeptides detected after enrichment of human serum

No.	Annotated Sequence	Master Protein Accessions	Theo. MH+ [Da]
1	LAGKPTHVnVSVVMAEVDGTcY	P01876	2348.128
2	VGQLQLSHnLSLVILVPQNLK	P05155-3	2314.348
3	TKPREEQFnSTFR	P01859	1640.801
4	FEVDSPVYnATWSASLK	P04114	1914.913
5	SWPAVGncSSALR	P02790	1405.656
6	SEGSSVnLSPPLEQcVPDR	P00734	2071.959
7	ALPQPQnVTSLLGcTH	P02790	1736.862
8	GLTFQQnASSMcVPDQDTAIR	P01871-2	2340.057
9	MVSHHnLTTGATLInEQWLLTTAK	P00738; P00739-2	2681.361
10	MDGASnVTcINSR	P08603	1425.608
11	SDFASnccSInSPPLYcDSEIDAELK	P02774-3	2994.234
12	MVSHHnLTTGATLINEQWLLTTAK	P00738; P00739-	2680.377

		2	
13	TPLTAnITK	P0DOX2	959.540
14	VVLHPnYSQVDIGLIK	P00738	1795.996
15	SPDVInGSPISQK	P08603	1342.685
16	NLFLnHSEnATAK	P00738; P00739-<br>2	1460.701
17	VIDFncTTSSVSSALAnTK	P04196	2016.945
18	EEQYnSTYR	P0DOX5	1190.497
19	NLFLnHSENATAK	P00738; P00739-<br>2	1459.716
20	EEQFnSTFR	P01859	1158.505
21	DTFVnASR	P05155-3	910.426
22	EEQFnSTYR	P01861	1174.503
23	SLGnVnFTVSAEALESQELcGTEVPSVPEHGRK	P01023	3543.690
24	HYTnSSQDVTVPcR	P0DOX2	1664.734
25	GVTSVSQIFHSPDLAIRDTFVnASR	P05155-3	2718.386
26	AnPTVTLFPPSSEELQANK	P0DOX8	2044.025
27	LSLHRPALEDLLLGSEAnLTcTLTGLR	P01876; P0DOX2	2964.581
28	ADGTVNQIEGEATPVnLTEPAKLEVK	P05090	2724.395
29	TAGWNVPIGTLRPFLnWTGPPEPIEAAVAR	P02788	3231.698
30	ADGTVNQIEGEATPVnLTEPAK	P05090	2255.102

31	VVAEGFDFANGInISPDGK	P27169	1950.945
32	IYPGVDFGGEELnVTFVK	P03952	1984.989
33	VVAEGFDFAnGInISPDGK	P27169	1951.929
34	VTVLGQPKAnPTVTLFPPSSEELQANK	P0DOX8	2866.521
35	IIVPLNNREnISDPTSPLR	P01591	2149.163
36	nLFLnHSENATAK	P00738; P00739-2	1460.701
37	mVSHHnLTTGATLINEQWLLTTAK	P00738; P00739-2	2696.371
38	IPcSQPPQIEHGTInSSR	P08603	2021.972
39	KVcQDcPLLAPLnDTR	P02765	1900.925
40	LcMGSGLnLcEPNNK	P02787	1707.753
41	VcQDcPLLAPLnDTR	P02765	1772.829
